# Secondary post-oncologic vulvar reconstruction – a simplified algorithm

**DOI:** 10.3389/fonc.2023.1195580

**Published:** 2023-06-20

**Authors:** Anna Amelia Caretto, Maria Servillo, Luca Tagliaferri, Valentina Lancellotta, Simona Maria Fragomeni, Giorgia Garganese, Giovanni Scambia, Stefano Gentileschi

**Affiliations:** ^1^ Dipartimento Universitario di Medicina e Chirurgia Traslazionale, Rome, Italy; ^2^ Casa di Cura Villa Stuart, BAC Center, Rome, Italy; ^3^ Fondazione Policlinico Universitario A. Gemelli IRCCS, Dipartimento Diagnostica per Immagini Radioterapia Oncologica ed Ematologia, Rome, Italy; ^4^ Fondazione Policlinico Universitario A. Gemelli IRCCS, Dipartimento Scienze della Salute della Donna, del Bambino e di Sanità Pubblica, Unità Ginecologia Oncologica, Rome, Italy; ^5^ Fondazione Policlinico Universitario A. Gemelli IRCCS, Dipartimento Scienze Della Salute Della Donna E Del Bambino E Di Sanità Pubblica, Unità di Chirurgia Plastica, Rome, Italy

**Keywords:** secondary vulvar reconstruction, gynecological cancer, vulvar reconstruction, vulvoperineal reconstruction, vulvar cancer, groin reconstruction, flap reconstruction

## Abstract

**Introduction:**

Surgical treatment is the gold standard of care for vulvar cancer and is burdened by a high risk of wound complications due to the poor healing typical of the female genital area. Moreover, this malignancy has a high risk of local relapse even after wide excision. For these reasons, secondary reconstruction of the vulvoperineal area is a relevant and challenging scenario for gynecologists and plastic surgeons. The presence of tissue already operated on and undermined, scars, incisions, the possibility of previous radiation therapy, contamination of urinary and fecal pathogens in the dehiscent wound or ulcerated tumor, and the unavailability of some flaps employed during the primary procedure are typical complexities of this surgery. Due to the rarity of this tumor, a rational approach to secondary reconstruction has never been proposed in the literature.

**Methods:**

In this observational retrospective study, we reviewed the clinical data of patients affected by vulvar cancer who underwent secondary reconstruction of the vulvoperineal area in our hospital between 2013 and 2023. Oncological, reconstructive, demographic, and complication data were recorded. The primary outcome measure was the incidence of wound complications. The secondary outcome measure was the indication of the different flaps, according to the defect, to establish an algorithm for decision-making.

**Results:**

Sixty-six patients were included; mean age was 71.3 ± 9.4 years, and the mean BMI was 25.1 ± 4.9. The mean size of the defect repaired by secondary vulvar reconstruction was 178 cm^2^ ± 163 cm^2^. Vertical rectus abdominis myocutaneous (VRAM), anterolateral thigh (ALT), fasciocutaneous V-Y (VY), and deep inferior epigastric perforator (DIEP) were the flaps more frequently employed. We observed five cases of wound breakdown, one case of marginal necrosis of an ALT flap, and three cases of wound infection. The algorithm we developed considered the geometry and size of the defect and the flaps still available after previous surgery.

**Discussion:**

A systematic approach to secondary vulvar reconstruction can provide good surgical results with a low rate of complications. The geometry of the defect and the use of both traditional and perforator flaps should guide the choice of the reconstructive technique.

## Introduction

Vulvar cancer is the fourth most common gynecological cancer and accounts for approximately 3%–5% of all malignancies of the female genital tract. Surgery is the standard treatment, involving a combination of radical vulvectomy with sentinel lymph node biopsy or, more frequently, inguinal lymph node dissection (1–3). Historically, vulvar cancer extirpation was considered a major surgery burdened by a high rate of postoperative complications. Over the years, to reduce morbidity without compromising survival, surgeons replaced the traditional radical en-bloc butterfly vulvectomy, described by Way in 1955 (4), with the so-called triple-incision technique using separate incisions over the inguinal ligaments (5). According to the tumor size and FIGO stage, several modifications of the vulvar dissection can be employed, such as radical local excision, extended vulvectomy, tumorectomy, or hemivulvectomy in early-stage disease and pelvic exenteration in patients with locally advanced disease. Chemoradiation treatment can be performed in a neoadjuvant or adjuvant setting. Despite the evolution in techniques, this aggressive resection is still associated with high rates of treatment-related morbidity and mortality (6). Postoperative wound complications remain one of the biggest problems in the care of these patients, with an incidence of 26%–85% (7), causing enormous physical and psychosocial problems, prolonged hospital stays, delays in the beginning of adjuvant therapies that can worsen oncologic outcome, as well as high healthcare costs. The most common postoperative short-term complications reported in the literature are wound breakdown, infection, lymphatic leakage, perineal disunion or cutaneous necrosis, and hematoma (8–10). Diabetes, jaundice, chronic renal failure, poor physical condition, advanced age, alcoholism, smoking, obesity, poor nutrition, and previous radiotherapy or chemotherapy are considered patient-related risk factors for short-term complications (11–14). Infection, wound dehiscence, and marginal necrosis of the perineum and groin are particularly frequent in overweight and obese patients (15–18). Reconstructive procedures associated with ablative surgery seem to be able to reduce the rate of wound dehiscence, vaginal introital stenosis, sexual dysfunction, and urinary problems. In fact, reconstruction reduces the tension of the sutures, fills the dead spaces, and provides healthy vascularized tissue to repair defects after extirpative surgery, increasing the possibility of uneventful wound healing. There is evidence in the literature that a systematic approach to vulvo-perineal reconstruction, including traditional and perforator flaps and considering the overall defect made of anatomical subunits, can improve results and reduce complications (19). One of the most challenging scenarios for post-oncologic vulvar reconstruction is that of secondary reconstructive procedures. Secondary reconstruction is reconstructive surgery performed after a previous extirpative vulvar surgery in which the defect had been repaired by advancement flaps and primary closure, by a graft, or by a flap. In clinical practice, this occurs when a patient has been operated on for vulvar cancer and repaired by primary closure or reconstruction, and wound breakdown or necrosis occurs or cancer relapses. Critical issues in these cases are the presence of previously operated and undermined tissues, the frequent bacterial contamination of the ulcerated recurrent tumor or of the wound dehiscence, the possibility of previous radiotherapy, scars, and the possible unavailability of some flaps if employed for previous reconstruction. There is not a defined reconstructive approach to these complex cases, and in this study, we analyzed our experience with secondary vulvar reconstruction and proposed an algorithm to decide the reconstructive strategy.

## Materials and methods

In this retrospective observational study, we reviewed the data collected in the clinical records and during the follow-up of all patients affected by vulvar cancer who underwent secondary vulvar reconstruction in our hospital between 2013 and 2023. Secondary vulvar reconstruction was intended as a reconstruction of a vulvoperineal defect following previous surgery for vulvar cancer extirpation associated with an advancement flap and primary closure or reconstruction. The study protocol was approved by the Institutional Review Board. The primary outcome measure of this investigation was the incidence of wound complications defined as dehiscence, infection, or flap edge necrosis. Secondary outcome measures were the surgical indications to establish the reconstructive technique according to the anatomical subunits involved in the defect. The variables of interest that we collected were the patients’ age, BMI, comorbidities, smoking habits, previous surgery, histological features, defect size, anatomical subunits involved in the defect, reconstructive techniques employed, size of the flap or graft, and wound complications. The wound complications considered were infection, dehiscence, and skin edge necrosis. To reduce the possibility of errors in the data detection and registration, we performed a double check whenever possible by comparing the data present in the digital clinical archive of our institution, which is available for outpatient and hospitalized patients, and in Redcap, a platform available in our hospital for online databases and surveys. The data collected were employed to build an algorithm to summarize our decisional process to decide the reconstructive technique when approaching secondary vulvar reconstruction.

### Statistical methods

The sample was described in terms of its clinical and demographic features using descriptive statistics techniques. Quantitative variables were described using the following measures: minimum, maximum, range, mean, and standard deviation. Qualitative variables were summarized in absolute and percentage frequency tables. The normality of continuous variables was checked using the Kolmogorov–Smirnov test and Hartley’s test for variance homogeneity.

## Results

Between 2013 and 2023, 66 patients underwent secondary vulvar reconstruction after another previous surgery for vulvar cancer extirpation. The patients’ ages ranged from 42 to 92 years, with a mean of 71.3 ± 9.4 years. BMI ranged from 18.8 to 42.9, with a mean of 25.1 ± 4.9. Six patients were smokers. Nine patients were obese and five were overweight. The defect repaired by secondary reconstruction was caused in 53 cases by a secondary extirpation for cancer relapse in patients previously operated for vulvar neoplasm and repaired by flap advancement and primary closure or by a graft or by a flap; we called this group of patients the “CR group.” In 13 cases, the indication for secondary vulvar reconstruction was a defect resulting from a wound breakdown or necrosis after recent ablative surgery for vulvar cancer repaired by flap advancement and primary closure, by a graft, or by a flap; we called this group of patients the “WD group.” Patients showing dehiscence of the sole inguinal incision for groin dissection were excluded. In both groups, the closure of the defect in the first surgery was performed by a plastic surgeon, either by advancement of the margins of excision and primary closure (so-called PAC—plastic assisted closure), a graft, or by flap. Considering all the patients, in 49 cases the previous surgery has been repaired by a primary closure, in 13 cases by a bilateral fasciocutaneous V-Y advancement flap (VY), in one case by a partial primary closure followed by secondary intention healing, in one case by a superficial circumflex iliac artery perforator (SCIP) flap, in one case by a skin graft, and in one case by a gluteal fold flap. A total of 50 patients had squamous cell carcinoma, 11 had Paget’s disease, four had vulvar intraepithelial neoplasia (VIN) + lichen sclerosus, and one had adenoidocistic carcinoma. The mean size of the surgical defect in the population examined was 178 cm^2^ ± 163 cm^2^. In the WD group, the mean size of the defect was significantly smaller than in the CR group (101 cm^2^ ± 93.5 cm^2^ *vs* 198 cm^2^ ± 172 cm^2^; WD group *vs* CR group, respectively, p <0.01). The other variables did not differ significantly. Dealing with the anatomical subunit, in all 66 patients, the defect involved the vulva and a variable amount of the perineal skin; in 32 patients, the defect also involved the mons pubis, and in 19 patients, the groin as well. In 15 patients, excision of pelvic viscera was performed: specifically, in two cases, anterior pelvectomy; in seven cases, posterior pelvectomy; and in six cases, complete pelvic exenteration. In all cases, we succeeded in achieving a complete closure of the defect.

The technique employed for secondary reconstruction was in 18 cases: vertical rectus abdominis myocutaneous (VRAM) flap; in 11 cases: anterolateral thigh (ALT) flap; in 11 cases: traditional fasciocutaneous VY flap; in seven cases deep inferior epigastric perforator (DIEP) flap; in five cases: superficial circumflex iliac artery perforator (SCIP) flap; in five cases: split thickness skin graft; in one case: Limberg flap; in three cases: gracilis myocutaneous flap; in two cases: pudendal thigh flap: in two cases: a perforator-based VY flap; and in one case: abdominal advancement flap (**Table 1**). In 29 cases, an adjunctive flap was employed in addition to the main reconstructive technique for wider defects. Specifically, 12 advancement flaps, eight traditional fasciocutaneous VY flaps, four ALT flaps, two SCIP flaps, one vastus lateralis flap, one pudendal thigh flap, and one profunda fermoris artery perforator (PAP) flap. In 45 patients, previous radiotherapy had been performed. After secondary vulvar reconstruction, in 53 patients the postoperative course was uneventful, while in nine patients wound complications and in four patients general complications occurred. We observed five cases of wound breakdown, one case of marginal necrosis of an ALT flap, and three cases of wound infection. Two patients developed urinary sepsis, and two patients developed deep vein thrombosis of the lower limb. In one case of wound breakdown and in the case of ALT flap marginal necrosis, we had to perform surgical debridement and direct closure. All other patients with complications healed by medical treatment without reoperation.

**Table 1:** The table shows the patient and defect characteristics and the reconstruction performed.

	Cancer status		Total (n = 66)
Demographic data	CR group (+) (n = 53)	WD group (*) (n = 13)	
Age (y)	71.8 ± 8.8	69.3 ± 11.8	71.38 ± 9.4
BMI (kg/m^2^)	25 ± 4.1	25.5 ± 7.2	25.1 ± 4.9
Underlying disease			
DM	4	1	5
Hypertension	11	3	14
Histology			
Squamous cell carcinoma	39	11	50
Paget	10	1	11
VIN + lichen	4	0	4
Adenoidocistic carcinoma	0	1	1
Reconstructive technique			
ALT	8	3	11
DIEP	4	3	7
VY (f, m, or p *)	2m -11f – 2p	1m	16
Limberg	0	1	1
Pudendal Thigh	1	1	2
SCIP	4	1	5
VRAM	15	3	18
Graft	5	0	5
Abdominal Advancement	1	0	1
Defect size	198 cm^2^ ± 172 cm^2^	101 cm^2^ ± 93.5 cm^2^	178 cm^2^ ± 164 cm^2^
Wound complication	5	4	9

DM, diabetes mellitus; VIN, vulvar intraepithelial neoplasia; ALT, anterolateral thigh flap; DIEP, deep inferior epigastric artery perforator flap; SCIP, superficial circumflex iliac artery perforator flap; VRAM, vertical rectus abdominis perforator flap. (*) f, fasciocutaneous; m, myocutaneous; p, perforator.

## Discussion

Recent trends in the surgical management of patients with vulvar cancer have emphasized the value of conservative operations, which help to preserve self-image and normal sexual function (20, 21). Despite this, about 50% of patients receive their first diagnosis at an advanced stage. The general condition of these patients is often debilitating because of tumor-related symptoms such as pain, itching, bad smell, lower limb lymphedema, and bleeding (22). Also, psychological conditions are very poor because of the awareness of having to suffer very intrusive treatments for body image and personal health. Specific counseling is mandatory on the issue that reconstruction can repair the defect and achieve local healing but cannot restore the original genital shape (23). For these reasons, the aggressive resections required for vulvar cancer extirpation are prone to frequent wound complications, prolonged hospitalization, and delayed adjuvant therapies. Relevant wound breakdown is often an indication for secondary reconstructive surgery because dead space under the wound is frequent and simple sutures are not effective. In these cases, secondary reconstruction can be urgent if radiation therapy is indicated (24). Furthermore, vulvar cancer has a high recurrence rate, with a reported 5-year local relapse rate of 21.4% (25), and local reexcision and reconstruction are frequently required, yielding acceptable survival rates of up to 51% at 5 years (26, 27). The cases requiring a secondary reconstructive procedure after a previous surgery present considerable challenges in their planning, execution, and management. Given the rarity of primary vulvar cancer, however, no study has analyzed a population of patients affected by vulvar cancer undergoing secondary vulvar reconstruction.

Our institution is a tertiary-level university teaching hospital with a very important department of gynecological oncology, receiving and treating a lot of patients affected by vulvar cancer from all over Italy. For this reason, we have overcome the scarcity of data available in the literature on the management of these patients and have developed a personal approach to reconstructive surgery planning and immediate postoperative period management. Our series demonstrates that it is possible to reconstruct difficult and extensive vulvar and perineal defects after previous surgery with a low rate of wound complications, following some principles. We must consider that about 85% of primary reconstructive surgery is accomplished in most centers employing a few traditional workhorse techniques: fasciocutaneous VY flaps and gluteal fold flaps for perineal defects; abdominal advancement flaps for mons pubis defects; and skin grafts for skinning vulvectomy in cases of intraepithelial Paget’s disease. The advancement flap and VY flap redistribute the tension over the suture but cannot eliminate it. Gluteal fold flaps can only repair defects of limited size and are not suitable for extended vulvectomy. Skin grafts in the vulvar area have a relevant risk of scarce engraftment due to poor wound healing typical of the female genital area affected by maceration and urinary and fecal pathogen contamination (28–30).

When approaching secondary reconstruction, in the case from a superficial defect of a skinning vulvectomy performed for intraepithelial Paget’s disease, the two most important reconstructive weapons are a skin graft and a thin SCIP flap, both of which are feasible even in the presence of previous radiation therapy (31). If previous grafting has been attempted and poor engraftment has occurred or cancer relapses, a SCIP flap is safer. This flap is often available for reconstruction because intraepithelial diseases almost never require groin surgery, and therefore the SCIP pedicles are intact (32, 33). If a SCIP flap is not available and a previous graft failed, the possibility of attempting debridement and a new graft in small defects or converting the defect to a full-thickness defect and repairing by a VY flap in bigger defects must be evaluated. When radical or extended vulvectomy has been performed during previous surgery and we are in the presence of a full-thickness defect, we must evaluate the location, size, and laterality of the defect and deal with flaps still available because they are not violated by the previous surgery.

**Figure 1:**
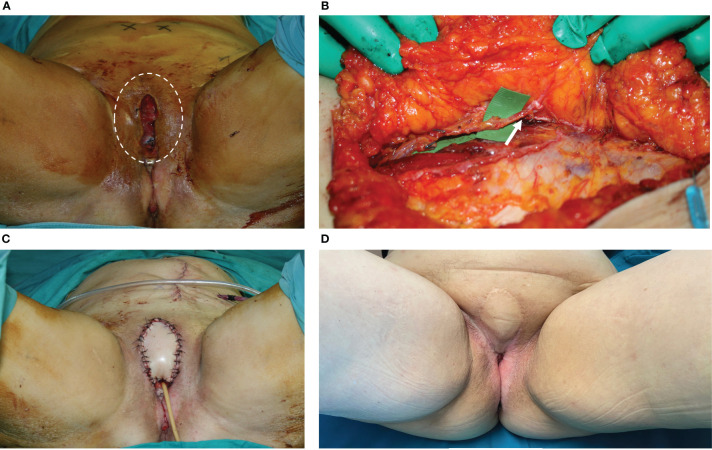
**(A)** This picture shows the wound breakdown of a patient affected by vulvar cancer who underwent radical vulvectomy and was repaired by advancement flaps and primary closure. The dehiscence involved the anterior perineum, in the area above the urethra, with a huge dead space under the edges of the cutaneous defect. The area of the dead space was indicated by the white circle. **(B)** The DIEP flap harvested from the left abdominal area. The white arrow indicated the perforator of the deep inferior epigastric artery. **(C)** The defect after debridement and DIEP flap inset, with closure of both the cutaneous defect and the subcutaneous dead space. The scar from the donor site was visible in the left abdominal area. **(D)** 12 months post-operative result, showing uneventful healing of the flap edges and normal scarring. The patient underwent adjuvant radiation therapy with no problems in the area repaired by the DIEP flap.

**Figure 2:**
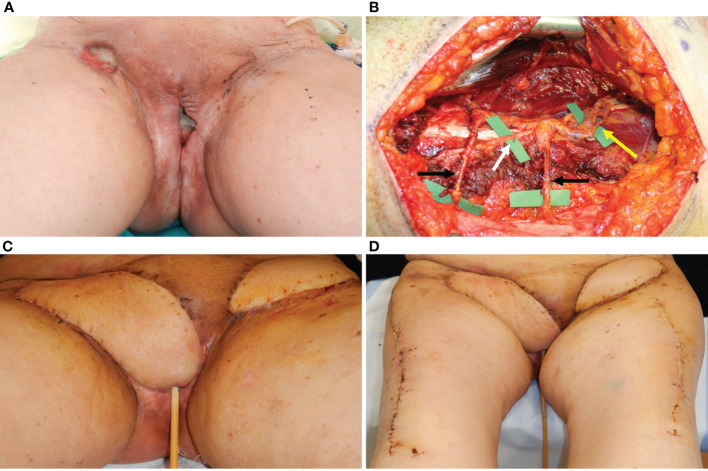
**(A)** A 72-year-old lady affected by vulvar cancer was operated on for vulvectomy and bilateral groin dissection for cancer relapse after a previous excision and radiation therapy. The patient showed two wound dehiscences: one in the right inguinal area and one in the upper perineal area. The two defects were communicating through a subcutaneous tunnel in the right bridge area. Moreover, in the left inguinal area, a skin resection had been planned to radicalize the previous groin dissection after definitive histology. **(B)** An ALT flap was planned from the right thigh to simultaneously repair the right groin and the perineal defect. Two myocutaneous perforators (indicated by the black arrows) were included, arising from the descending branch of the lateral circumflex iliac artery (indicated by the yellow arrow). The motor nerve (white arrow) to the vastus lateralis muscle was completely spared. An ALT flap was also planned in the contralateral thigh to repair the left inguinal defect **(C)** Three-week post-operative result, after bilateral ALT flaps show normal healing of perineal and inguinal scars. **(D)** ALT donor sites, closed primarily 3 weeks after surgery.

**Figure 3:**
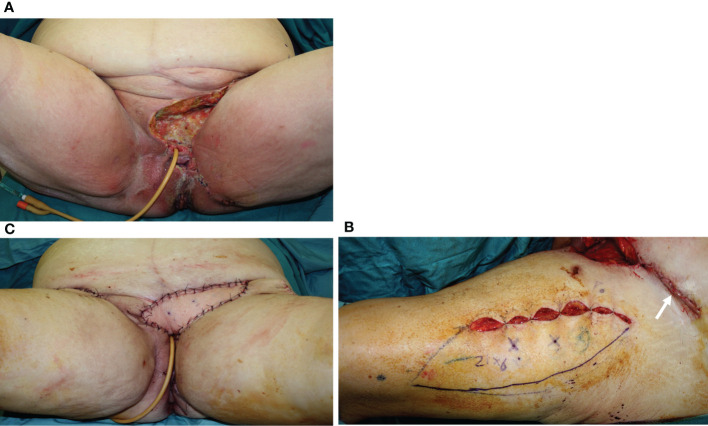
**(A)** A 75-year-old patient showing a defect involving the upper perineum, mons pubis, and left groin after radical vulvectomy repaired by advancement flaps and wound breakdown. **(B)** An ALT flap planned on the left thigh with a 21 × 6 cm. skin paddle. The white arrow indicated the scar of the abdominal advancement flap, joined with the dehiscent incision for groin dissection, like an abdominoplasty scar. **(C)** The flap inset after debridement of the defect and secondary reconstruction.

In cases of anterior defects, involving the area above the urethra with or without mons pubis, if the groin is not included in the defect, a DIEP or VRAM flap is the best option (**Figure 1**); if the groin is a part of the defect, an ALT flap is our choice (**Figures 2**, **3**). SCIP was often excluded in these cases because previous inguinal surgery on the groins often damaged its pedicles.

In the presence of a defect of small/medium size in the middle and posterior perineum, if VY flaps are available, a traditional fasciocutaneous VY or a perforator based VY flap is our choice. This flap is safe even in the case of previous radiation therapy.

**Figure 4:**
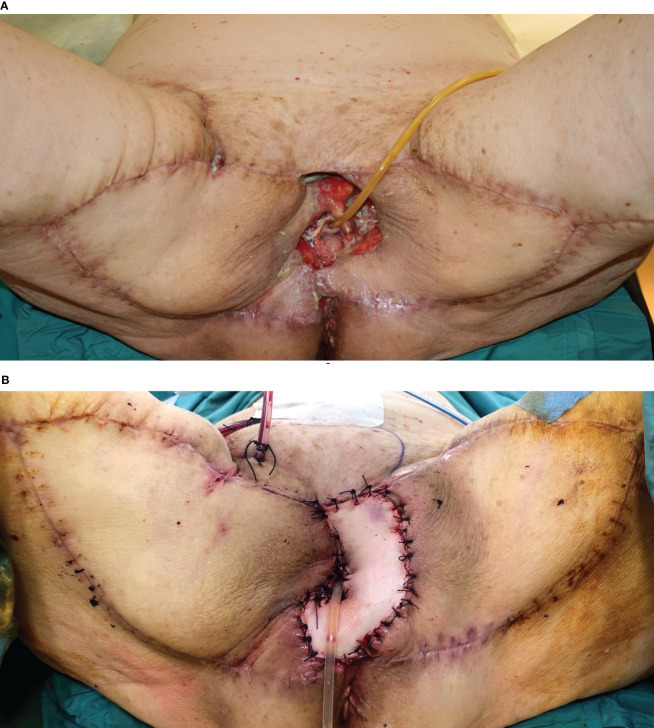
**(A)** A 74-year-old patient underwent, 1 month before, a radical vulvectomy for vulvar cancer and was repaired by bilateral fasciocutaneous VY advancement flaps. Relevant dehiscence with a central defect was evident already 13 days after surgery. The upper edge of the defect showed huge dead space under the mons pubis. **(B)** Secondary reconstruction of the defect after debridement with VRAM flap harvested from the right abdominal area. The skin paddle repaired the perineal defect, while the rectus muscular belly filled the huge dead space in the mons pubis area. The postoperative course of this patient was uneventful, and she could begin radiotherapy after 60 days since the first procedure.

**Figure 5:**
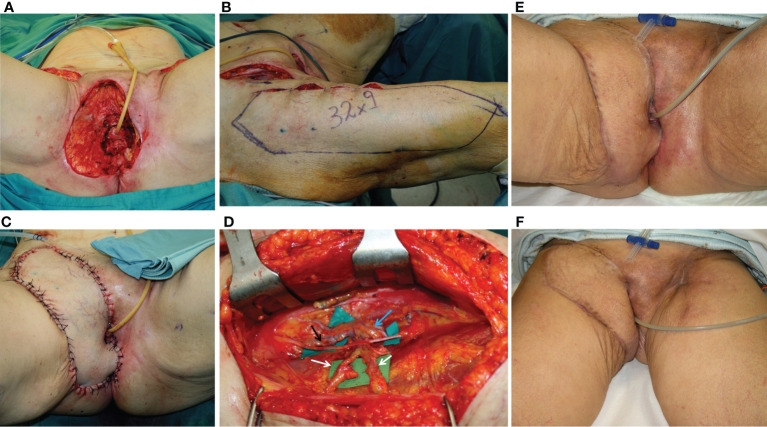
**(A)** Defect of the entire right perineal area, including the mons pubis and right groin, after extirpation of recurrent vulvar cancer. **(B)** ALT flap harvested on the right thigh with a 32 × 9 cm skin paddle. **(C)** ALT flap inset for secondary vulvo-perineal and right groin reconstruction. **(D)** A detail of the pedicle of the flap, made up of two perforators (indicated by white arrows) arising from the descending branch of the lateral circumflex iliac artery (indicated by the blue arrow). The motor nerve for the vastus lateralis muscle (black arrow) was completely spared. **(E, F)** Late postoperative results, showing uneventful wound healing.

In the presence of a defect of small/medium size in the middle and posterior perineum, when VY flaps have already been employed for previous surgery, or in the case of a bigger defect, involving the whole perineum with or without the mons pubis VRAM flap (**Figure 4**) or ALT flap (**Figure 5**) is the best option. The ALT flap can be unilateral or bilateral, according to the defect geometry.

**Figure 6:**
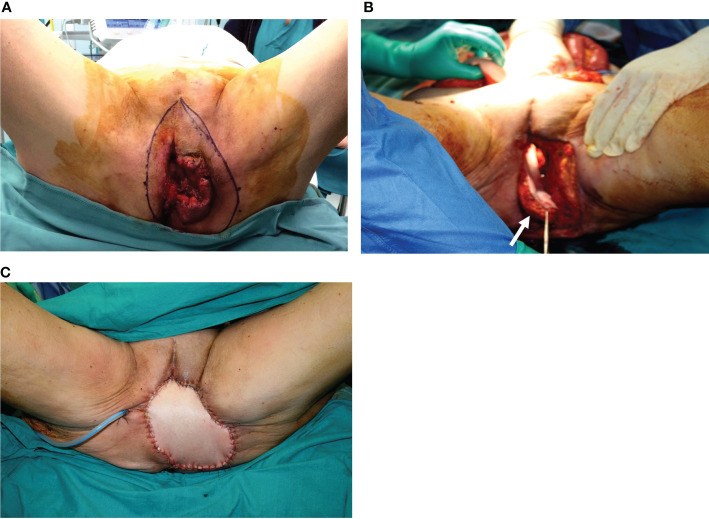
**(A)** Massive vulvar cancer relapse after surgery and radiation therapy. Previous surgery consisted of a vulvectomy, repaired by flap advancement and primary closure. **(B)** Complete pelvic exenteration was performed, and the skin paddle of the VRAM flap (indicated by the white arrow) passed through the pelvis and the pelvic floor to reach the perineal defect. **(C)** Inset of the flap completed.

**Figure 7:**
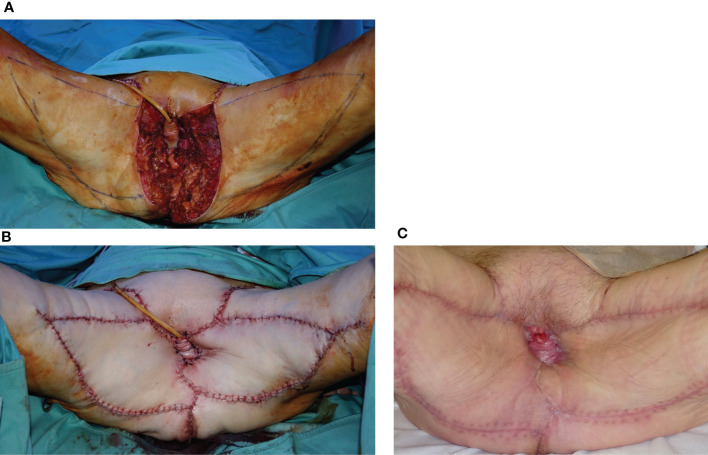
**(A)** Posterior pelvectomy performed for recurrent vulvar cancer after previous surgery and radiation therapy. **(B)** The defect was repaired by bilateral advancement VY flaps. **(C)** Late postoperative results, showing uneventful wound healing.

If an anterior or complete pelvectomy is performed, a VRAM flap with an intrapelvic course is our first option (**Figure 6**) because it can fill the dead space, close the pelvic floor to the bowel, and provide a huge skin paddle. In the case of posterior pelvectomy, bilateral VY flaps are feasible (**Figure 7**) if available and in the presence of medium-small defects, while a VRAM flap is the alternative.

**Figure 8:**
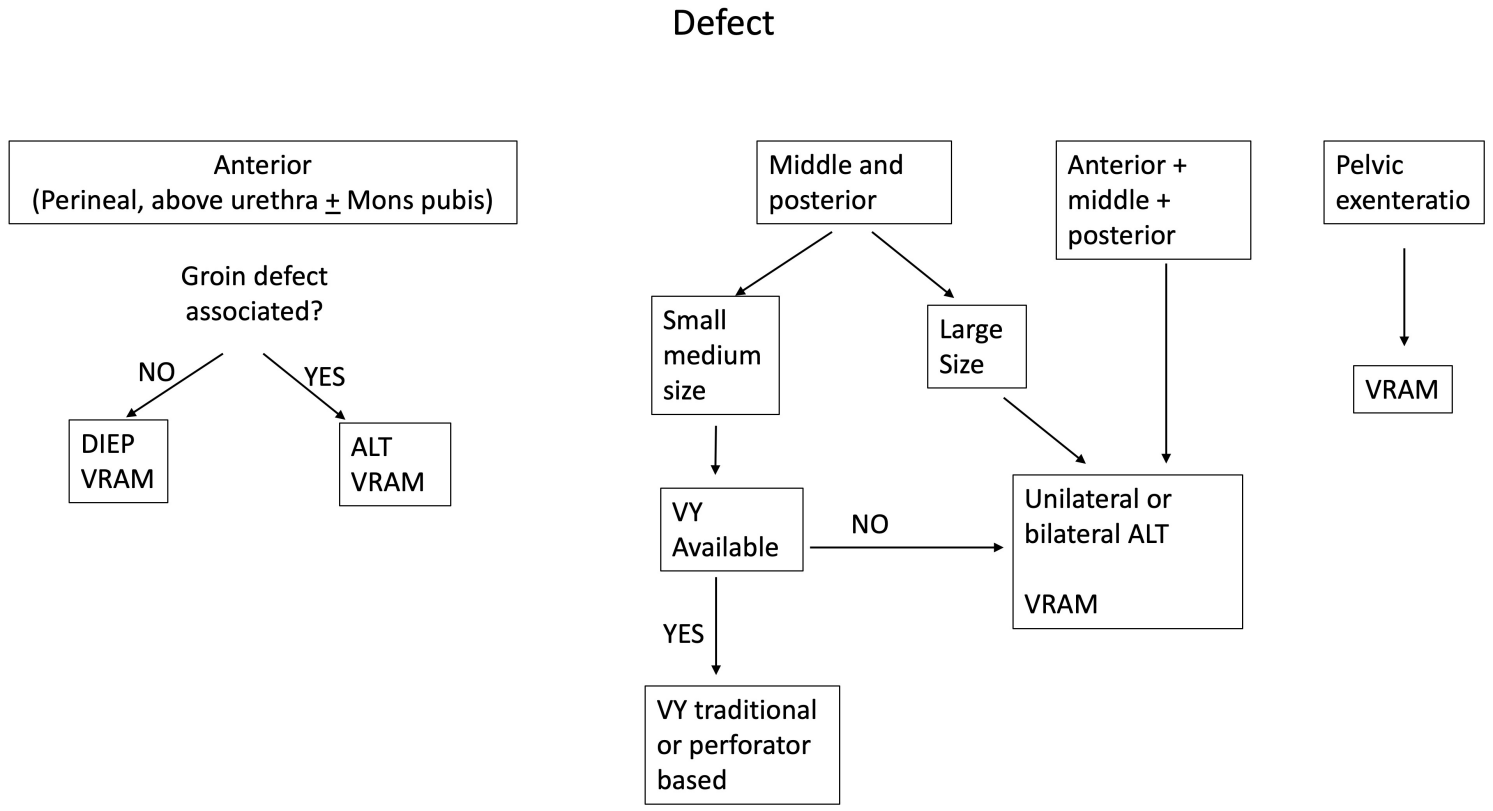
The figure shows the algorithm that we propose to help with the decision-making process for secondary vulvar reconstruction.

Analyzing the types of flaps employed for reconstruction in our series, VRAM, ALT, VY, and DIEP were used in 47 patients, accounting for 71% of the cases and representing the most useful flaps for secondary reconstruction in our experience. It must be noticed that two are traditional flaps and two are perforator flaps. This is consistent with current literature, where perforator-based flaps have been emerging as a trend in primary perineal reconstruction (34–40), and we confirm this trend for secondary reconstruction in the algorithmic approach that we implemented (**Figure 8**).

The low complication rate observed in our series must also be related to the close cooperation between plastic surgeons and gynecologist oncologists in the pre-operative planning, during the surgery, and in the post-operative period, following a strict protocol already reported (41).

Our approach, based mainly on the analysis of the anatomical subunits involved in the defect rather than on simple size criteria, and the inclusion of both traditional and perforator flaps, is consistent with other algorithmic approaches already published in the literature for primary perineal reconstruction (19, 40, 42–46).

The main limitations of this study may be considered the absence of a control group treated using a different approach, the retrospective and single-center design. Nevertheless, the evaluation was carried out on a good number of patients, considering the low prevalence of vulvar cancer in the population.

In conclusion, a strict rational approach, including a rigid postoperative system of patient management, can provide good reconstructive results with a low incidence of complications, even in the challenging scenario of secondary vulvar reconstruction.

## Data availability statement

The datasets presented in this article are not readily available because Not available. Requests to access the datasets should be directed to Stefano Gentileschi, stefanogentileschi@gmail.com.

## Ethics statement

Written informed consent was obtained from the individual(s) for the publication of any potentially identifiable images or data included in this article.

## Author contributions

Methodology: AC, GS and SG. Software: AC, MS and SG. Validation: SG. Formal analysis: AC and SG. Investigation: AC, SF, GG, and SG. Data curation: AC and SG. Writing—original draft preparation: AC, LT and VL. Supervision: SG. All authors contributed to the article and approved the submitted version.
